# Sexual function after Anchorsure repair of sacrospinous ligament suspension in patients with pelvic organ prolapse: a prospective multicenter study

**DOI:** 10.1038/s41598-026-52719-w

**Published:** 2026-05-11

**Authors:** Neda Ahmadian, Elnaz Rastkar, Elaheh Amiri, Parvin Bastani, Reza Sattarpour

**Affiliations:** Department of Gynecology, Women’s Reproductive Health Research Center, Tabriz University of Medical Sciences, Tabriz, Iran

**Keywords:** Pelvic organ prolapse, Sacrospinous ligament suspension, Anchorsure system, Female sexual function index, Sexual dysfunction, Quality of life, Diseases, Health care, Medical research, Urology

## Abstract

Sacrospinous ligament suspension (SSLS) is widely used to restore apical support, yet the impact of SSLS using the Anchorsure system on postoperative sexual function remains insufficiently characterized. This prospective multicenter observational cohort study enrolled 115 women with symptomatic apical pelvic organ prolapse undergoing SSLS with the Anchorsure system at two teaching hospitals in Northwest Iran between 2022 and 2024. Sexual function was assessed preoperatively and at 3‑month follow‑up using the Persian‑validated Female Sexual Function Index (FSFI). 115 women were enrolled and completed baseline FSFI, and 104 (90.4%) completed the 3‑month assessment. Mean FSFI total score increased from 19.15 ± 1.94 preoperatively to 21.84 ± 3.98 postoperatively (*p* < 0.001, Cohen’s d = 0.88), with statistically significant improvements in desire, arousal, orgasm, satisfaction, and pain domains. Lubrication improved only minimally (mean Δ = 0.03 ± 0.71, *p* = 0.024, d = 0.04). Age and body mass index were not significantly associated with postoperative FSFI scores (*p* > 0.05). Sacrospinous ligament suspension with the Anchorsure system was associated with significant short‑term improvements in sexual function in women with apical pelvic organ prolapse, particularly in arousal, satisfaction, and pain reduction. These findings support SSLS with Anchorsure as a native‑tissue surgical option for patients who prioritize sexual health outcomes or are not candidates for more extensive abdominal procedures.

## Introduction

Human sexuality is a fundamental aspect of individual well-being and quality of life (QoL). Menopause-related changes in estrogen and androgen levels negatively impact domains of sexual function, including vaginal lubrication, arousal, orgasm frequency, and dyspareunia risk^1,2^. Additionally, ageing-associated physiologic changes like vaginal atrophy, decreased elasticity, and diminished blood flow complicate these effects and contribute to sexual dysfunction in postmenopausal women^3^. However, because the topic is delicate, studying older women’s sexuality can present challenges, especially in conservative cultures. Systematic reviews and meta-analyses document that 40–60% of women with moderate-to-severe pelvic organ prolapse report sexual dysfunction, including decreased desire, arousal difficulties, dyspareunia, and anorgasmia^4^. Among women with apical prolapse specifically, sexual dissatisfaction has been reported in up to 67% of cases, particularly in those over age 50^5–7^. This condition can manifest with a variety of symptoms, including pelvic pressure, urinary or fecal incontinence, and sexual dysfunction^8^.

POP can significantly impair a woman’s sexual function through various mechanisms. The physical symptoms of prolapse, such as vaginal bulging and pelvic pressure, can cause discomfort and interfere with sexual activity^9^. Furthermore, the psychological distress associated with POP, including body image concerns and feelings of embarrassment, can negatively impact sexual desire and arousal^10,11^. Studies have indicated that a substantial proportion of women with POP experience female sexual dysfunction, characterized by difficulties with desire, arousal, orgasm, and pain during intercourse^4^. The presence of urinary or fecal incontinence, often co-occurring with POP, can further exacerbate sexual dysfunction due to fear of leakage during sexual activity^10^.

Sacrospinous ligament suspension surgery (SSLS), an autologous tissue repair technique, has emerged as a solution for restoring vaginal apical support^12^. This procedure involves anchoring the vaginal vault to the sacrospinous ligament, a robust pelvic structure, to provide enhanced support^13^. Multiple studies of sacrospinous ligament suspension have reported postoperative improvements in sexual function or sexual activity scores. These improvements are generally attributed to restoration of apical support and reduction of prolapse‑related symptoms, which in turn decrease discomfort during intercourse and improve body image and sexual confidence^14–17^. In contrast, some research indicates no significant change in overall sexual function after SSLS^18,19^. While less common, some studies have reported no significant change or even a decline in sexual function, including increased dyspareunia, after SSLS^20–22^. Since the FDA issued warnings regarding surgical mesh use in (POP) surgery, there has been renewed interest in techniques that utilize patients’ own tissues^23^. While sacrospinous ligament suspension has been established as a durable surgical approach for apical prolapse management, published studies specifically examining the impact of the Anchorsure system on postoperative sexual function remain limited. Prior studies of traditional SSLS techniques have reported conflicting outcomes, with meta-analyses documenting dyspareunia rates as high as 14.36%^24^. The purpose of this study was to investigate the impact of Anchorsure SSLS on patients’ post-operative sexual function in all subscales beyond dyspareunia.

## Method

### Study design

This study was a prospective observational research focused on evaluating sexual satisfaction among women undergoing sacrospinous ligament suspension surgery (SLSS). The target population included women candidates for this surgery at two teaching hospitals in northwest Iran between 2022 and 2024. Multiple approaches for apical prolapse are offered, including: abdominal sacrocolpopexy with mesh, traditional transvaginal SSLS with uterosacral ligament suspension, and obliterative procedures in select cases. All patients selected for SSLS with Anchorsure in this study chose this approach after informed consent discussions that included discussion of alternative techniques, their respective durability, anatomical outcomes, mesh-related risks, and sexual function considerations. The study assessed sexual satisfaction both before and after the surgery to evaluate its impact on sexual function. Inclusion criteria were women with symptomatic pelvic organ prolapse (POP-Q stage ≥ 2 in the apical compartment or symptomatic stage 1 apical prolapse) undergoing planned SSLS as determined by urogynecologist consultation, with documented symptoms (pelvic pressure, bulging sensation, or sexual dysfunction), age ≥ 18 years, and written informed consent. Exclusion criteria included active genital infection, congenital or structural genital anomalies, inability to provide informed consent, or pregnancy. SSLS was the primary apical support procedure in all included cases, and women undergoing concomitant hysterectomy, hysteropexy, colpopexy, mesh augmentation, or other major reconstructive procedures at the index operation were excluded. A minority of participants had undergone a prior hysterectomy before the index SSLS; this variable was recorded as a baseline characteristic and included in univariate analyses of postoperative FSFI scores. Patients were included if SSLS was the primary surgical procedure for apical prolapse management. Concurrent anterior or posterior compartment repairs (when anatomically indicated) were permitted and documented. However, patients undergoing concurrent major reconstructive procedures beyond simple anterior/posterior point fixation (e.g., anterior colporrhaphy, posterior colporrhaphy, or biological graft augmentation) were excluded.

Patients were assessed for preoperative sexual activity status and the Female Sexual Function Index (FSFI) score. Sexual activity was defined as participation in any form of sexual contact with a partner (including but not limited to penetrative intercourse) within the 4 weeks prior to baseline FSFI administration. FSFI was developed by R. Rosen et al.^25^ and adapted into Persian by Babakhani et al. in 2018 for use among postmenopausal women, demonstrating high reliability (Cronbach’s alpha > 0.80) and strong intraclass correlation coefficients (> 0.96)^26^. This multidimensional, self-reported questionnaire consists of 19 items that evaluate female sexual dysfunction across six domains: desire, arousal, lubrication, orgasm, satisfaction, and pain. Each domain consists of specific items scored on a Likert scale ranging from 0 to 5, with higher scores indicating better sexual functioning. FSFI questionnaires were administered preoperatively (within 2 weeks prior to surgery) and at a standardized postoperative timepoint of 3 months following surgery, allowing adequate tissue healing and functional recovery by trained fellows in urogynaecology who administered and assisted participants in completing the questionnaire. Pain during intercourse (dyspareunia) was assessed using the pain domain of the FSFI, which includes two items: frequency of pain during or after vaginal penetration and level of discomfort associated with penetration. A combined pain subscale score of 0 indicates the absence of dyspareunia, while a score > 0 indicates dyspareunia. Because a domain score of zero typically reflects the absence of recent sexual activity rather than maximal dysfunction, domain-level analyses were prespecified to be conducted among women who reported at least some sexual activity in the preceding 4 weeks at each time point. Sexually inactive women completed the FSFI, but their domain scores were summarized descriptively and not pooled with sexually active women for domain-level analyses.

The study protocol was approved by the Institutional Review Board and the Ethics Committee of Tabriz University of Medical (Reference Number: IR.TBZMED.REC.1401.811). All procedures were conducted in accordance with the Declaration of Helsinki. Written informed consent was obtained from all participants prior to enrollment and data collection.

### Surgical technique

The SSLS surgery aims at vaginal apex stabilization using native tissue from the patient. During this procedure, a urogynecologist creates an incision through the vaginal wall to anchor the apex of the vagina to the sacrospinous ligament located along the pelvic bones and sacrum. This technique is classified as native tissue repair (NTR), utilizing the patient’s tissues rather than synthetic materials. Permanent or absorbable sutures are used, which eventually form scar tissue around them to provide long-term support for the vaginal structure^27^. The sacrospinous ligament’s robust fibrous structure provides a stable anchor point for vaginal apex suspension. By attaching the prolapsed vaginal vault to this ligament, surgeons restore the natural axis of the vagina, aligning it vertically over the levator plate. This reorientation redistributes intra-abdominal pressure away from weakened pelvic floor structures, thereby reducing the risk of recurrence^28,29^.

All procedures in our study were performed by experienced urogynecologists (two surgeons across two institutions) with > 5 years of SSLS experience using the Anchorsure system. Standardized perioperative protocols were employed at both institutions, including identical patient positioning, vaginal preparation, anesthesia type (general or regional), and postoperative care. We used the Anchorsure method, a type of normal tissue repair, to fixate ligaments^30^. The Anchorsure System is a pelvic floor repair tissue-fixing anchor system with a thin, straight applicator device explicitly engineered for safe anchor placement at the sacrospinous ligament. The Anchorsure procedure first involves identifying the pararectal area on the posterolateral aspect of the pelvis. The rectum is moved away from the surgical site to prevent injury, and the vaginal mucosa is dissected from the rectovaginal septal plane^31^. The sacrospinous ligament is palpated digitally to create space for the Anchoring tool. In all cases, a unilateral right‑sided SSLS was performed. Two non‑absorbable Anchorsure sutures were placed into the right sacrospinous ligament approximately 1.5–2 cm medial to the ischial spine, and these sutures were used to suspend the vaginal apex. While NTR has regained prominence due to concerns about mesh-related complications, the Anchorsure System avoids the use of large mesh sheets^32^.

### Statistical analysis and sample size

The study was designed as a prospective multicenter cohort with a fixed 24-month recruitment window at two high-volume urogynecology centres, and no formal a priori sample size calculation was performed. Based on expected recruitment, a target of approximately 115 participants was considered feasible and sufficient to detect clinically relevant changes in FSFI total scores. A post hoc calculation indicated that the final sample of 115 women provided approximately 80% power (α = 0.05, two-sided) to detect a 2.5-point within-subject difference in FSFI total score, corresponding to a large effect size (d ≈ 0.75). The data analysis was conducted using IBM SPSS Statistics, Version 22 (IBM Corp., Armonk, NY, USA; available at: https://www.ibm.com/products/spss-statistics). Before analysis, the normality of data distribution was verified using the Kolmogorov-Smirnov test, which determined the selection of appropriate statistical tests for subsequent analyses. Descriptive statistics were calculated to characterize the study variables, including means, medians, quartiles, and standard deviations. For the primary comparison of sexual satisfaction scores before and after surgical intervention, paired statistical tests were employed. Specifically, the Paired T-test was utilized for normally distributed data, while the Wilcoxon signed-rank test was applied for non-normally distributed data. Visual representations, including charts and graphs, were also used to illustrate data distributions and relationships among the variables of interest. The significance threshold was consistently set at *p* < 0.05 throughout all statistical analyses, providing a standard benchmark for interpreting results.

## Results

During the enrollment period, 142 women were evaluated for SSLS at the two institutions. Of these, 115 women met the inclusion criteria and agreed to participate (enrollment rate: 81.0%). Twenty-seven women were excluded: 8 (30%) declined participation, 6 (22%) had POP-Q stage 1 apical prolapse without symptoms, 5 (19%) had congenital genital anomalies, 4 (15%) were planning pregnancy within 12 months, and 4 (15%) had active genital infections that required treatment prior to surgical candidacy. All 115 enrolled participants completed the preoperative FSFI. Postoperative FSFI completion rate at 3 months was 104 of 115 (90.4%); 11 participants (9.6%) were lost to follow-up (unable to be contacted at the postoperative assessment interval). The study included 115 participants aged 29 to 76 years, with a mean age of 52.74 ± 11.95. The participants’ reproductive history ranged in gravida from 0 to 13, with a mean of 4.40 ± 2.297, and in parity from 0 to 10, with a mean of 3.69 ± 1.988. Also, the mean BMI was 27.34 ± 4.18. Diabetes Mellitus was present in 11.0% of the participants. Additionally, 8.5% had a previous hysterectomy, and 3.4% had undergone previous stress urinary incontinence surgery. Table 1 demonstrates the basic characteristics of the participants. All participants underwent Sacrospinous ligament suspension (SSLS). The Pelvic Organ Prolapse (POP) stages varied across compartments (see Table 2). In the anterior compartment, stage 2 was most common (63.3%), followed by stage 3 (16.5%). In the posterior compartment, stage 2 was predominant (75.0%), followed by stage 1 (10.7%). The apical compartment was mainly stage 2 (58.4%), followed by stage 3 (21.2%).

**Table 1:** Basic characteristics of the patients.

Age	Mean/ Percent	SD*	*p*-value^¥^
	52.7391	11.95319	0.617
Gravidity	4.40	2.297	
Parity	3.69	1.988	
NVD* delivery	3.47	2.116	
BMI*	27.3352	4.17682	0.513
Menopause (yes)	52.2%		0.088
DM (yes)	11.5%		0.611
Neurologic disease (yes)	0.9%		0.517
Previous hysterectomy (yes)	8.8%		0.348

SD: standard deviation/ NVD: Normal Vaginal Delivery/ BMI: Body Mass Index.

¥: correlation with post-operative female sexual index score.

**Table 2:** Staging of pelvic organ prolapse based on compartment (values expressed as percentage [%] of participants according to POP-Q classification)

Compartment		Stage (% of participants)				
		0	1	2	3	4
Anterior compartment		1.8	13.8	63.3	16.5	4.6
Posterior compartment		1.8	10.7	75.0	8.0	4.5
Apical compartment		0	15.9	58.5	21.2	4.4

Among the 115 women included, 94 (81.7%) were sexually active at both time points, and 21 (18.3%) reported no sexual activity in the preceding 4 weeks. Total FSFI scores increased significantly in the sexually active group from 19.84 ± 3.92 preoperatively to 22.54 ± 4.25 postoperatively (*p* = 0.002), with a modest positive correlation between pre‑ and postoperative scores (*r* = 0.312, *p* = 0.006). In contrast, no significant change was observed in non‑sexually active women (12.86 ± 3.88 vs. 13.18 ± 4.11, *p* = 0.294). In the overall cohort, total FSFI improved from 19.15 ± 1.94 to 21.84 ± 3.98 (*p* = 0.004), with a corresponding correlation coefficient of *r* = 0.269 (*p* = 0.010). Across domains, sexually active women demonstrated significant postoperative improvements in desire (3.02 ± 0.94 to 4.05 ± 1.21, *p* < 0.001), arousal (2.82 ± 0.91 to 4.25 ± 1.31, *p* < 0.001), lubrication (3.40 ± 1.03 to 3.43 ± 1.10, *p* = 0.036), orgasm (3.35 ± 0.88 to 3.63 ± 0.94, *p* < 0.001), satisfaction (3.55 ± 1.12 to 4.42 ± 1.41, *p* < 0.001), and pain (3.75 ± 1.64 to 4.67 ± 1.42, *p* < 0.001), with strong within‑subject correlations for lubrication (*r* = 0.784), orgasm (*r* = 0.769), satisfaction (*r* = 0.792), and pain (*r* = 0.603; all *p* < 0.001). In contrast, non‑sexually active women showed a statistically significant but smaller increase in desire (2.65 ± 0.83 to 3.25 ± 1.01, *p* = 0.012, *r* = 0.442, *p* = 0.028), with no significant changes in arousal, lubrication, orgasm, satisfaction, or pain (all *p* > 0.05). Preoperatively, 32 of the 94 sexually active women (34.0%) reported dyspareunia. At 3‑month follow‑up, dyspareunia had completely resolved in 15 of these 32 women (46.9%), while 4 women (12.5%) reported persistent but less intense pain; the remainder were no longer sexually active. Among women who were pain‑free and sexually active before surgery and remained sexually active postoperatively, none developed de novo dyspareunia.

When all participants were analyzed together, domain scores mirrored these patterns, with significant improvements in desire, arousal, lubrication, orgasm, satisfaction, and pain (all *p* < 0.001), and consistently strong correlations between pre‑ and postoperative domain scores, particularly for lubrication (*r* = 0.784), orgasm (*r* = 0.748), and satisfaction (*r* = 0.765; all *p* < 0.001). Table 3 compares these domains pre- and post-operatively in detail. The overall FSFI score demonstrated a significant positive correlation (*r* = 0.269, *p* = 0.010) between pre-operative and post-operative measurements, reflecting absolute improvements across the cohort despite individual heterogeneity. Strong positive correlations emerged for lubrication (*r* = 0.784, *p* < 0.001), orgasm (*r* = 0.748, *p* < 0.001), and satisfaction (*r* = 0.765, *p* < 0.001), indicating that women with higher preoperative scores in these domains maintained relatively higher postoperative scores. These results suggest that surgical intervention had a positive impact on most aspects of sexual function. Additionally, no significant correlations were found between postoperative FSFI scores and age (*p* = 0.617) or BMI (*p* = 0.513). Also, we found no significant differences in postoperative FSFI between diabetics (*p* = 0.611), menopausal women (*p* = 0.0886), or women with prior hysterectomy (*p* = 0.348) on sexual function outcomes.

**Table 3:** Female Sexual Function Index (FSFI) domain scores before and after surgery in sexually active and non‑sexually active women.

		Pre-operative Mean ± SD*	Post-operative Mean ± SD	*p*-value	Correlation coefficient (*r*)	*p*-value (correlation)
	Group					
Total FSFI	SA	19.84 ± 3.92	22.54 ± 4.25	0.002	0.312	0.006
	NSA	12.86 ± 3.88	13.18 ± 4.11	0.294	0.210	0.335
	Total	19.15 ± 1.94	21.84 ± 3.98	0.004	0.269	0.010
Desire	SA	3.02 ± 0.94	4.05 ± 1.21	< 0.001	0.511	< 0.001
	NSA	2.65 ± 0.83	3.25 ± 1.01	0.012	0.442	0.028
	Total	2.93 ± 0.89	3.93 ± 1.32	< 0.001	0.487	< 0.001
Arousal	SA	2.82 ± 0.91	4.25 ± 1.31	< 0.001	0.566	< 0.001
	NSA	2.31 ± 0.70	2.45 ± 0.88	0.228	0.302	0.185
	Total	2.56 ± 0.75	3.89 ± 1.40	< 0.001	0.521	< 0.001
Lubrication	SA	3.40 ± 1.03	3.43 ± 1.10	0.036	0.784	< 0.001
	NSA	–	–	–	–	–
	Total	2.86 ± 0.68	2.91 ± 0.82	< 0.001	0.784	< 0.001
Orgasm	SA	3.35 ± 0.88	3.63 ± 0.94	< 0.001	0.769	< 0.001
	NSA	3.12 ± 0.66	3.25 ± 0.82	0.118	0.622	0.003
	Total	3.32 ± 0.82	3.59 ± 1.00	< 0.001	0.748	< 0.001
Satisfaction	SA	3.55 ± 1.12	4.42 ± 1.41	< 0.001	0.792	< 0.001
	NSA	3.02 ± 1.21	3.35 ± 1.43	0.082	0.589	0.006
	Total	3.89 ± 1.35	4.75 ± 1.76	< 0.001	0.765	< 0.001
Pain	SA	3.75 ± 1.64	4.67 ± 1.42	< 0.001	0.603	< 0.001
	NSA	–	–	–	–	–
	Total	2.77 ± 1.07	1.85 ± 1.11	< 0.001	0.603	< 0.001

FSFI = Female Sexual Function Index; SD = standard deviation; SA = Sexually active; NSA = not Sexually active. Higher scores indicate better function except for pain, where higher scores indicate less pain.

## Discussion

### Summary of findings

In our cohort of 115 women undergoing sacrospinous ligament suspension (SSLS) with an anchor based system for apical pelvic organ prolapse, short term sexual function improved significantly across most FSFI domains among women who remained sexually active. Total FSFI scores increased meaningfully at 3 months, driven by improvements in desire, arousal, orgasm, satisfaction, and pain, whereas lubrication changed only minimally despite statistical significance. Within**-**subject correlations indicated that women with higher baseline lubrication, orgasm, and satisfaction tended to maintain relatively better postoperative scores in these domains. The absence of de novo dyspareunia and the high rate of dyspareunia resolution among women with preoperative pain suggest that, in the early postoperative period, SSLS with an anchor system is more likely to relieve pain than to generate new sexual dysfunction symptoms.

### Interpreting our results

When evaluating the impact of POP surgery on sexual function, it is essential to consider surgical approaches and their respective outcomes. Although abdominal sacrocolpopexy (ASC) is widely regarded as the anatomical gold standard for apical prolapse repair, concerns about mesh-related complications and variable subjective outcomes, including sexual function, have challenged its use in some settings^33^. Systematic reviews and meta-analyses indicate that ASC offers superior anatomical durability compared with SSLS but may not consistently translate into better quality-of-life or sexual function outcomes^4,20,22,34^. In our previous trial, both ASC and SSLS groups exhibited significant postoperative improvements in pelvic floor distress measures, including POPDI-6, CRADI-8, UDI-6, and overall PFDI-20 scores^35^. Notably, the ASC group achieved significantly greater reductions in total PFDI-20, POPDI-6, and UDI-6 scores compared to the SSLS group, whereas CRADI-8 scores showed no significant difference between the groups. Patient satisfaction rates were high and comparable across both groups. Beyond these two established approaches, several alternative techniques have been investigated. Amrute et al. reported a 2.5-year follow-up analysis of patients who underwent transvaginal repair of total pelvic organ prolapse with a single polypropylene mesh and concluded that transvaginal mesh repair is a safe and efficacious option^9^. However, a recent Cochrane review by Yeung et al. indicated that transvaginal absorbable mesh and biological grafts showed no clear advantage over native tissue repair in terms of prolapse awareness, repeat surgery, or recurrent prolapse, with uncertain effects on bladder outcomes and quality of life ^37^. Specifically, transvaginal mesh repair was not superior to native tissue repair in subjective success or safety at 36 months, with similar rates of serious adverse events, although anatomical outcomes were somewhat improved with mesh^36^.

Concerns about recurrence and adverse outcomes further complicate the choice of surgical technique. Ishchenko et al. found that POP recurrences typically occurred 12–36 months after surgical treatment, with a higher likelihood of recurrence in patients who underwent surgery using their own tissues compared with synthetic implants^37^. Similarly, Shatkin-Margolis and Pauls noted that the evidence on transvaginal mesh repair remains conflicting, with some data suggesting a potential negative impact on women’s quality of life and sexual function^7^. With respect to SSLS specifically, Zhang et al. reported in their meta-analysis that SSLS was associated with a threefold higher dyspareunia rate compared with ASC (14.36% vs. 4.67%)^24^. Nevertheless, the authors also found that, when considering factors such as mesh erosion, operative time, gastrointestinal complications, haemorrhage, and wound infections, SSLS may be the preferable option. The elevated incidence of dyspareunia associated with SSLS warrants attention due to its impact on sexual function; however, literature specifically addressing recently developed anchoring devices, such as the Anchorsure system, rather than the conventional SSLS technique itself, remains limited.

Turning to the present study, our findings on overall sexual outcome improvement align with earlier studies demonstrating symptom and quality-of-life improvement after SSLS. Stratified analysis demonstrated that postoperative gains in sexual function were driven primarily by women who remained sexually active, whereas those who remained sexually inactive showed low and unchanged FSFI totals. Among sexually active women, desire, arousal, orgasm, and satisfaction all improved significantly, and the pain domain showed a substantial increase in score, consistent with a clinically meaningful reduction in dyspareunia. In contrast, lubrication scores among sexually active participants increased only minimally (3.40 ± 1.03 to 3.43 ± 1.10; *p* = 0.036), despite a strong pre-to-post correlation, indicating that the statistically significant change corresponds to a very small effect and is unlikely to be clinically important. These domain-level findings are consistent with reports that POP surgery most strongly affects arousal, satisfaction, and pain, whereas lubrication is more closely related to age and hormonal status. Corroborating these observations, studies using validated questionnaires, such as the Pelvic Floor Distress Inventory (PFDI) and Pelvic Floor Impact Questionnaire (PFIQ), have similarly shown reductions in symptom scores and improvements in overall well-being^38,39^.

From a procedural standpoint, the SSLS technique’s reliance on autologous tissue minimizes foreign body reactions, though scar tissue formation remains critical for durability^13,24^. In this regard, Senturk and Dogan O demonstrated the safety and feasibility of SSLS under local anaesthesia using the Anchorsure system in elderly patients with significant comorbidities^40^. Notably, our findings differ from the meta-analysis by Zhang et al. reporting elevated dyspareunia rates following SSLS (14.36% with SSLS vs. 4.67% with ASC)^24^. In contrast, we observed significant pain reduction (*p* < 0.001) rather than pain escalation. Several factors may explain this discrepancy. The Anchorsure system employs minimally invasive anchor placement, which may avoid excessive tissue trauma compared to traditional suture-passing techniques. Moreover, Our study is among the first to assess sexual outcomes with the newer Anchorsure technology. Nevertheless, the absence of de novo dyspareunia at 3 months among women who remained sexually active should be interpreted cautiously, because late‑onset pain related to scar remodelling or recurrent prolapse may emerge beyond the early postoperative window.

## Clinical implications and counselling recommendations

Our preceding findings of broad FSFI improvement with particularly gains in arousal, satisfaction, and pain, alongside the absence of de novo dyspareunia carry several practical implications for preoperative counselling. While most women in our study, reported improved sexual function following SSLS, it is important to consider the procedure’s potential impact on all aspects of sexual well-being. Previous studies have focused on dyspareunia as the main post-operative concern; yet, other domains of sexual function besides pain also improved significantly following SSLS in the current study. More broadly, Sexual function after prolapse surgery is influenced not only by anatomic correction but also by psychological and relational factors, including body image, fear of pain, and partner dynamics, as highlighted in previous studies of women undergoing POP repair^11^. Carlin et al., for example, revealed multiple factors influencing sexual activity and desire for children following surgery, with fear of incontinence during sexual activity emerging as a significant concern among sexually inactive participants^11^. Recent research has also begun to explore the relationship between clitoral anatomy and sexual function following vaginal surgery for POP, including SSLS; Bowen et al. found that better overall postoperative sexual function correlated with a larger clitoral glans width and thickness among sexually active women^10,41^. Pre-existing sexual dysfunction is an important consideration in surgical counseling, as baseline sexual function may influence postoperative outcomes. Women with severe preoperative sexual dysfunction related to prolapse symptoms may experience symptomatic improvement post-surgery if anatomical correction addresses the underlying etiology. Conversely, sexual dysfunction with psychological or relationship-based etiologies may persist postoperatively despite anatomical correction^4,10,11^. Our findings should therefore be interpreted in the context of comprehensive preoperative counseling that addresses expectations, pre-existing sexual dysfunction, and the potential for persistent or non-anatomic contributors to sexual difficulties.

Based on our findings, practitioners counselling women with apical prolapse should consider the following:

(1) Sexual outcome expectations: Women prioritizing sexual health outcomes should be informed that SSLS with Anchorsure is associated with improvement across multiple sexual function domains, particularly arousal and satisfaction, with a marked reduction in dyspareunia. These improvements were sustained at 3-month follow-up. Women with minimal preoperative sexual dysfunction may benefit from explicit discussion of anticipated satisfaction improvements, whereas those with pre-existing dysfunction should be counselled that gains may be more modest, particularly if non-anatomic factors contribute to their symptoms.
(2) Comparative effectiveness and patient selection: While abdominal sacrocolpopexy remains the anatomical gold standard^35^, SSLS with Anchorsure offers a minimally invasive alternative with favorable sexual function outcomes and may be particularly suitable for, women with significant surgical comorbidities unable to tolerate extended abdominal surgery, those prioritizing rapid recovery and native-tissue repair techniques, patients with prior abdominal surgery complicating reoperation, and women in whom durability is balanced against tissue preservation.
(3) Preoperative evaluation and expectation-setting: Comprehensive preoperative evaluation should include sexual history, assessment of pre-existing sexual dysfunction, which is a known predictor of poorer postoperative outcomes, relationship status, and realistic expectation-setting. Women with minimal preoperative sexual dysfunction may benefit from explicit discussion of anticipated satisfaction improvements. Surgeons should also highlight the Anchorsure system’s precision in anchor placement and avoidance of mesh-related complications, which may provide psychological reassurance to patients concerned about mesh erosion or other long-term adverse effects.

### Limitations and direction for future research

To the best of our knowledge, our study is one of the few worldwide that examines sexual function in patients following the anchor system SSLS. Because of the relatively large sample size and the inclusion of patients from two hospitals that are representative of the general population, our study’s findings should be more broadly applicable. However, our study has some limitations. As acknowledged in the protocol, the 3-month postoperative assessment window, while adequate for immediate functional recovery, does not capture longer-term sexual function outcomes beyond the immediate postoperative period. Recurrent prolapse or vaginal fibrosis may emerge at 12–24 months, potentially affecting sexual function. Our study lacks a comparison group (e.g., abdominal sacrocolpopexy, transvaginal mesh repair, or conservative management). Consequently, we cannot definitively attribute sexual function changes to SSLS specifically versus general improvement following any POP intervention, surgical recovery, or placebo effect. The heterogeneity of surgical approaches (isolated vs. combined anterior/posterior repair) may cause variability in sexual function outcomes. While this reflects real-world clinical practice in the management of complex POP, future prospective studies comparing isolated SSLS to combined reconstruction approaches would further clarify the relative impact of each procedure on sexual function.

Additionally, the FSFI is a self-reported questionnaire subject to recall, social desirability, and response shift biases. In the Iranian cultural context, where discussing sexual function remains sensitive, participants may have underreported sexual dysfunction preoperatively or overreported satisfaction postoperatively due to perceived physician expectations or cultural stigma. The cohort age range spans pre-, peri-, and postmenopausal women with different hormonal statuses and baseline sexual function profiles. While menopause status was not a significant predictor in univariate analysis (*p* = 0.088), this may reflect insufficient statistical power to detect modest interactions.

## Conclusion

Our study demonstrated that sacrospinous ligament suspension using the Anchorsure system is associated with short‑term improvement across multiple FSFI domains in women with apical pelvic organ prolapse, with particularly notable gains in arousal and pain reduction and minimal change in lubrication. These findings support SSLS with Anchorsure as a native‑tissue surgical option for women who prioritize sexual health outcomes or are not candidates for abdominal sacrocolpopexy, while noting the need for longer‑term comparative studies incorporating both anatomical and psychosocial measures.

## Data Availability

The datasets generated and/or analyzed during the current study are not publicly available due to the hospital’s policy, but are available from the corresponding author on reasonable request.
